# Crossing paths: historical and philosophical perspectives on cancer and diabetes classifications

**DOI:** 10.1007/s40656-026-00729-2

**Published:** 2026-06-10

**Authors:** Sarah Yvonnet, Karin Tybjerg

**Affiliations:** 1 Medical Museion, Department of Public Health Sciences, University of Copenhagen, Copenhagen K, Denmark; 2 Novo Nordisk Foundation Center for Basic Metabolic Research, Faculty of Health and Medical Sciences, University of Copenhagen, Copenhagen K, Denmark

**Keywords:** Classification, Cancer, Diabetes, Biomarkers, Diagnosis

## Abstract

No one is perfect, and this is particularly true of disease classifications. When diseases are classified according to different dimensions (topography, symptoms, morphology, aetiology, etc), some characteristics of the diseases are prioritised while others are downplayed. Moreover not all diseases are equally well characterised by a given dimension, and especially complex, chronic diseases such as cancer and diabetes have proved difficult to classify because of their heterogeneity. Their classification created tensions historically and still present challenges today. In this article we juxtapose the difficulties of classifying diabetes and cancer historically with the challenges arising in modern classifications of the two diseases. We trace the history of classifying cancer and diabetes, and find that while cancer proved difficult to classify based on its physiological features, diabetes could not readily be situated anatomically and they were classified according to different dimensions: cancer as patho-anatomical and diabetes as patho-physiological. We then discuss the limitations that come with the adoption of these classifications and how they are currently being challenged by new scientific developments. We show that the use of molecular markers to reclassify diabetes 2 and cancer allows their characterisation to cross paths i.e. to move across the classificatory dimensions that had been prioritised historically. Reopening down-prioritised dimensions comes at a price, though, and, we outline challenges as well as opportunities faced by type 2 diabetes and cancer classification. Finally, we argue that the combination of philosophical and historical analyses is well suited to cast light on classificatory tensions and challenges.

## Introduction

The ability to classify and diagnose disease is a matter of great importance and consequence to medical practice (Smart, 2014; Bowker & Star, 2000). In clinical medicine, diagnoses and classifications for instance allow medical practitioners to assign a patient to a given specialty and offer the most fitting treatment and prognosis. In medical research they help to establish the inclusion and exclusion criteria in clinical trials, delineate the circumstances of drug approval, or conduct epidemiological studies (Jutel, 2009). And in health administration and public health, they play a central role as different disease categories give access to different services and status such as insurance reimbursement or sick leave, and for patients they shape patient identities and communities around certain diseases (Jutel, 2009). Last, classifications and diagnoses are not just important for clinical, research and societal understandings of the disease, and for connecting between them (Kutschenko, 2011). They also serve to make diseases intelligible across time (Rosenberg, 2003; Tybjerg, 2023). Indeed, medical knowledge depends on comparing to past and current cases and this requires stability in naming and classifying across time.

No one, however, is perfect and this is especially true for classifications and categorisations of disease. In the last decade, particularly complex chronic and non-transmissible diseases have been challenging our ways of classifying diseases (Broadbent, 2009; Fuller, 2018). These are highly heterogeneous, multicausal diseases and they manifest both locally and systemically, which makes it difficult to establish satisfactory disease classifications and categories. In this article, we especially focus on the challenges that arise because classificatory systems cannot capture all dimensions of diseases because it is not straightforward which dimension to prioritise when defining and classifying them.

Diseases can be defined according to several dimensions, which may in turn be used for classification purposes. The philosopher of medicine Nordenfelt identified four dimensions of diseases that have been used across different classificatory systems: topography, morphology, aetiology, and function (Nordenfelt, 2013). Staphylococcus pneumonia, for instance, is localised in the tissues of the lung (topography), characterised by an inflammation (morphology), explained by the presence of staphylococci bacteria (aetiology) and described in terms of diminished breathing capacities (function)^1^. However, being able to define all four dimensions of a disease is not always as easy as in the case of staphylococcus pneumonia, nor do all the dimensions make equal sense for defining, differentiating and investigating particular diseases. Last, and this is of particular importance in this paper, the diseases cannot be classified equally according to them all within the same system - one is prioritised above the others.

Dimensions thus have to be prioritised relative to each other for classificatory purposes. Such classificatory prioritisations are a product of both historical contexts and how easily a disease is characterised by the given dimension. For instance, current classifications of diabetes and cancer focus on two different dimensions. In the ICD-11, cancers are classified mainly according to their anatomical site of origin (topography), whereas diabetes is classified according to the lack of glucose regulation (functional). These classifications are now facing pressing challenges. Indeed, current diabetes and cancer classification and categories have been acknowledged as being too broad, and this has motivated the search for fine-grained disease categories. As a way to achieve this, current research in both fields is mobilising molecular and genetic information as a main way to stratify patients. The issue, however, is not just one of categories being too broad, so stratifying existing categories will not fully resolve it. It also—as we shall show—involves reclassifications or a reopening of dimensions downplayed by the current classificatory system.

To understand the possibilities and limitations of today’s classificatory system, we argue it is helpful to consider classificatory history and how classifications and conceptions of diseases have developed in conjunction with each other. The current classifications were shaped by historical conceptions and practices and these conceptions remain enfolded in classifications as prioritised dimensions. We may indeed understand the grand historical shifts in medical focus from symptoms of deviation from a healthy balance, to pathological lesions and physiological deviations (e.g. Temkin, 1977; Jewson, 2005; Boenink & van der Molen, 2024) as shifts in the prioritisation of different dimensions. In this way, the dimensions of classification, like Nordenfelt’s, may be historicized. Juxtaposing the tensions emerging in early classifications of cancer and diabetes with those arising in today’s, will show how medical researchers today may be seen to grapple with integrating present and historical conceptions when classifying disease.

Thus, in this article, our aim is double. (1) We will trace the history of classifications of cancer and diabetes showing how the diseases became classified according to different dimensions. In both cases, the historical perspective shows how the priorisation of one dimension over another delimited and focused the understanding of the diseases. And (2) we will analyse how modern attempts to reclassify the diseases challenge these historically established conceptions as well as re-open dimensions of the disease that were present in the past.

We focus on cancer and diabetes as both are at the centre of recent attempts to refine disease categories, and while the stratification of cancer has attracted philosophical attention (Green et al., 2022; Plutynski & Laplane, 2023; Tinland et al., 2024), diabetes is less studied. Moreover, they represent paradigmatic examples of how different dimensions have been prioritised in the classificatory systems, with cancer being classified according to its topographic dimension (i.e. the anatomical site where the tumour originated) while diabetes is classified by its functional dimension (i.e. first excessive urination and later sweet urine or blood glucose levels).

In the first section, we discuss classification and how history shapes current medicine through it. The second section offers a historical trajectory of how cancer and diabetes have been investigated and classified. We show how the diseases historically did not lend themselves to be classified according to the same dimensions, and we suggest a distinction between a patho-anatomical dimension (related to the topographic dimension) and patho-physiological dimension (related to the functional dimension). We use a different terminology as our historicized categories of patho-anatomical are broader than Nordenfelt’s topography and function. Our patho-anatomical dimension includes lesions both in specific organs and tissues as well as localized symptoms; and our patho-physiological dimension includes symptoms of excess or lack as well as function.^2^ We then shift to a more philosophical analysis of how current research, investigating molecular classification, challenges the dimensions of disease that had become prioritised in the course of history. We consider cancer and diabetes broadly in the historical section, but when dealing with the modern approaches to the diseases, we focus on breast cancer and type 2 diabetes. We thus follow the historical trend towards a more detailed categorisation of disease, and this narrowing of focus partly reflects the increasing stratification of cancer and diabetes, and partly draws on some of the most relevant and well-studied examples—breast cancer and type 2 diabetes.

What we find is that recent attempts to sub-classify cancer and diabetes both orient themselves towards the dimensions that have historically been downplayed and challenge the established classification system of the two diseases. Cancer classification has already for some years explored a patho-physiological dimension, and diabetes classification has recently begun to explore a patho-anatomical dimension. With the increasing use of biomarkers *the diseases may be seen to move across classifications*. We thus offer a classificatory history of cancer and diabetes: How they first became unrelated as they were understood through different dimensions, and how they now begin cross paths with current movements in the fields of breast cancer and type 2 diabetes researching the dimension prioritized by the other. Two diabetes researchers introduced a recent article linking type 2 diabetes to certain cancer types by stating that: “From time to time, workers in apparently unrelated fields look sideways to discover that others are working on the same problem from a different angle. This happened recently when investigators in diabetes and cancer found that they were exploring much the same territory” (Smith & Gale, 2010, p. 1541). Our account does not claim the two fields are the same territory, but rather explores how they first became unrelated through being classified according to different dimensions, and now have started to approach each other as researchers are crossing the patho-anatomical and patho-physiological dimensions. Past classificatory prioritisations delimit how we understand disease, but re-visiting downplayed dimensions may open past insights.

I. Classifications carry past notions of disease and prioritise dimensions of disease

It is well established that understandings and classifications of disease change over time as they are shaped by their historical contexts (Rosenberg, 2002). We argue, however, that we should pay more attention to the conserving effect of classifications. As stated earlier, classifications and categories make it easier to draw on past cases of disease and this is essential for developing medical knowledge. Indeed we often hang onto imperfect classifications because stability is valued above precision (Bowker & Star, 2000, pp. 76, 112). As a case in point, the overall structure of classification in *The International Classification of Disease* (ICD) has remained relatively constant over the last hundred years allowing its stable use over time. Even in earlier periods of the 18th and 19th, when classificatory systems changed more frequently, continuity was valued. Extensive labour was put into copious notes connecting old and new classifications together by detailing how a disease was classified in the several previous systems. Historical conceptions of disease thus left their imprint on classifications because classifications are constructed on the basis of past cases; and they remained constant despite new aspects of a disease emerging. As Bowker and Star formulate it about the ICD: “the list folded its history in on itself”. This means that classifications while conserving the highly necessary ability to draw on past knowledge also create a form of conceptual inertia, because historical classifications frame the disease in the present.

The classificatory system serves to make the landscape of disease easier to survey by prioritising and delimiting what we need to consider—for better or worse. Often the dimension by which a disease has historically been detected or recognized is prioritised when defining the disease. This could be a lesion in a particular body part or tissue, which we denote patho-anatomical; or it could be a symptom of unbalance or a change in physiological or biochemical levels, which we denote patho-physiological. The prioritised dimension allows the disease to be easily diagnosed and at the same time frames how it is to be further investigated. On the flipside, classifications limit understanding because a given classification makes some aspects of the disease and some paths of investigation or research appear less obvious. As we shall see, current classifications of cancer and diabetes are shaped by 19th and 20th century approaches to the diseases—but not by the full range of ways in which the diseases were investigated and conceptualised in these periods. Rather they are shaped by the dimensions that were prioritised in the classificatory systems.

These prioritised dimensions also shape how a disease is related to other diseases. Classificatory systems cannot bring out all relations between diseases equally well. If breast cancer is classified as a disease of the breast, it highlights its relations to other diseases of the breast. Classifying cancer as neoplasia—growth of abnormal cells—or by tumour types, highlights its relation to other forms of cancers or tumours of the same type. And a classification of cancer as a wound or an excess of fluids, might relate it to wounds for instance from venereal disease or to diseases such as diabetes or cholera which were historically characterised by increased flow. The dimensions prioritised in classifications thus establish sameness and difference highlighting or downplaying different relations across the landscape of disease - relations that may reemerge if diseases are investigated according to down-prioritised dimensions again.

History is important for analysing disease classifications because the classifications import history into present medicine. It is therefore not a coincidence that a recent study by Nick Binney (2024), which argues for the epistemic role of history for understanding current medicine, deals with classification. He deals with thyroid cancer, and shows how an array of sub-divisions of the cancer ends up producing misleading categories, as the clinically important question of malignancy does not align with the resulting categories. He does not conceptualise it as such, but his study shows how the development of categories and classifications is *path dependent*, as the divisions made in the past and the order in which they were made, shape the resulting categories. So although Binney argues that history is important more broadly, the fact that it proves itself particularly useful to understand a classificatory issue is telling. Classifications can hide dimensions down-prioritised in the past, and to reveal them again we need to historically unbraid the classificatory system.

The prioritizations between dimensions have often been conceptualised as mirroring the needs of different users. This is the case both in early classifications and today’s scholarship. William Farr, author of an early classification of causes of death, notes for instance that clinicians, pathologists, physiologists and lawyers have different classificatory needs: “The medical practitioner may find his main divisions of diseases on their treatment as medical or surgical; the pathologist on the morbid action or product; the anatomist or physiologist on the tissues or organs involved; the medical jurist, on the suddenness or the slowness of the death; …” (Farr, 1856, p. 8). This is quoted in the ICD-6—a predecessor of the current system of disease classification adopted in 1948—which goes on stating that “the various titles will represent a series of necessary compromises between classifications based on aetiology, anatomical site, age and circumstance of onset, …” (World Health Organization, 1949, pp. XII–XIII). In recent work on classification, Lara Keuck has also noted that the broad classes, which are sometimes deemed unsatisfactory, allow them to serve different users—e.g. administration, patients, and hospitals; and she distinguishes these broad classifications from narrower classifications employed in research (Kutschenko, 2011). In our account we focus, however, less on how classifications serve different users and more on how they have framed diseases across time. We also note that although research is often engaged in refining classifications, the broader clinical classifications still shape approaches to diseases in research—by choice of classificatory dimensions and by defining the group of sufferers from whom researchers may source samples or data and to whom clinical applications of the research might be directed.

Summing up, the investigation of classificatory history supplies an important key to the study of classification. This is because history is enfolded in classifications and when particular dimensions of diseases are prioritised, other dimensions are downplayed. Classifications connect to and preserve past understandings of disease, but they also close off or hide connections to them.

II. Historical classifications: Branching into patho-anatomical and patho-physiological dimensions

By tracing the classification history of cancer and diabetes we will show that classifications were shaped by and—in turn shape—conceptualisations of the diseases. At different points in history, the two diseases in different ways generated tensions in the classificatory systems. This was not only because they combine local and general aspects, but also because the ways they were understood and investigated in different historical periods did not fit equally well with all dimensions, and the two diseases ended up on different classificatory paths. We recognise the inevitable difficulties of tracing diseases over centuries which involves an element of “identifying” diabetes and cancer across historical contexts. While diabetes presents a recognisable entity throughout the history of medicine, the understanding of the disease changed drastically from 18th to 21st century and diabetes was mixed with other forms of excessive urinary flow. Cancer, tumours and cancerous ulcers are even harder to view as a unified group as ulcerations were difficult to distinguish from other lesions e.g. from STDs. Historians of cancer in the early modern period and 19th centuries however maintain that cancers were viewed as a distinct disease albeit one that was understood on the disturbed flows until far up into the 19th century (Skuse, 2015; Arnold-Forster, 2021). What we do here has under all circumstances less to do with imposing a modern-day conception on historical classifications, and more to do with the opposite movement. How can we understand tensions in modern classifications through past ones?

- Late 18th and early 19th centuries

18th and 19th century classificatory systems, or nosologies, were primarily based on symptoms and physiological characteristics. Drawing on observations by the bedside, they are often classified on the basis of paired symptoms such as excess or lack of heat, tension, breathing or flow. In François Boissier de la Croix de Sauvages’ (1706–1767) nosology, (*Pathologia methodica* 1752) diabetes was classified under “flows that are not of blood or excrement” and described as excessive and frequent urination^3^. Tumours and cancers on the other hand were not presented as a unified disease phenomenon and they were found in two classes, first under skin conditions and secondly under wasting, constitutional or general disease (*cachexia*). The Scottish physician William Cullen (1710–1790) attempted to simplify the system, by using fewer main classes with his *A methodical system of nosology* (1769/1808). This divided diseases in Fevers, Nervous, Wasting/General and Local, and here diabetes was classified as a nervous spasm in “the natural functions” and grouped together with other diseases of excessive flow such as cholera and diarrhoea. Cancer sat in a class of Local diseases i.e. diseases of particular body parts, and the cancers were differentiated according to whether they formed hard or soft tumours or ulcers. Moving into the 19th century, the British surgeon and apothecary John Mason Good published *A physiological system of nosology* (1817) which criticised Cullen’s class of Local diseases for being too mixed and unsystematic. He classified diseases according to physiological systems such as the Digestive, Respiratory, Blood-related, Nervous, Sexual, Secretion- and Exhaustion-related systems and Chance, and he thus allowed more classes. He classified diabetes under Secretion and Exhaustion and subgrouped it under urinary disorders characterising it by excessive flow, sweet taste and emaciation. Tumours also fell in this class, as the swelling was seen as a sign of a lack of balance between secretion and exhaustion, and tumours were subdivided according to their feel, composition and structure as well as their localization in bones, veins, testes, breasts and scrotum. Good additionally listed cancer in another class—Blood Diseases—as morbid blood vessels—and he noted that cancer is not always local, but can be constitutional (Good, 1817, p. 251).

What emerges here is that diabetes was relatively stably described by its symptoms of excessive flow of urine, sweet urine and emaciation. At the time, it fitted unproblematically into the systems that classified diseases under different physiological symptoms and functions—even if it figured both under diseases related to Secretion and to Nerves. Cancers and tumours were spread across the classifications and did not constitute a unified disease; they were classified both under wounds and swellings; they were variously characterised as diseases of the surface, as local diseases or as constitutional diseases; and they were divided both according to the character of the tumours and according to their locations.

(b) 19th century and statistical classification

In the course of the 19th century new ways of understanding and investigating disease through particularly pathological anatomy and physiological experiments developed, and offered new ways of characterising diabetes and cancer. The second half of the 19th century also saw the development of the main structure of the ICD system, whose original purpose was not so much to classify diseases, but rather causes of death for statistical purposes. The work towards an international classification started with lists compiled by two statistically minded doctors – the British William Farr and the Swiss Jacob Marc d’Espine. They had different emphases in their classifications. Farr divided diseases into epidemic and sporadic diseases being interested in health and hygiene, and d’Espine divided them into acute and chronic being interested in aetiology and duration; they both however introduced location and anatomical pathology into the subclassifications of their systems. Farr privileged pathological lesions noting that causes of death such as dropsy or paralysis were out of favour with pathologists, because they could be symptoms of underlying lesions in several different organs - and thus not fit categorising under one organ. He therefore urged “observers to refer specifically to the primary organic lesion whenever it can be satisfactorily determined” (Farr, 1856, pp. 6–7). D’Espine for his part classified according to different natures of diseases, but focused on the patho-anatomical dimension in his subclassification of the sporadic diseases, which he divided into Constitutional disease (*cachectici*) involving several organs, and Local diseases (*monoorganici*) disturbed a particular organ (Farr, 1856, p. 14). As for cancer and diabetes, Farr, classified cancers as Constitutional disease and sub-categorised them according to the type of tumour, while diabetes was listed as a Local disease of the kidneys. In D’Espine’s system both diabetes and cancer were classified as chronic diseases, but diabetes was listed under diseases of the blood or of sugar/albumin in the urine, while cancer was sub-classified according to its anatomical site (d’Espine reprinted in Lewes, 1988). When it came to amalgamating Farr’s and D’Espine’s systems into a single one, a compromise list underwent several revisions, and was not settled till the International Statistical Institute in 1893 agreed on a system based on Jacques Bertillon’s *Classification of Causes of Death*, which was subsequently widely adopted and published (American Health Association, 1899; Moriyama et al., 2011). Here, both diabetes and cancer figured under constitutional disease, but cancers were clearly sub-divided according to its anatomical sites, as in d’Espine, while the classification of other diseases followed Farr and divided them under diseases of the heart, lungs etc. The patho-anatomical dimension was thus forefronted both in the sub-categorisation of cancers and in the overall classes.

So, while cancer at the close of the 19th century was still classified as a constitutional disease, it was increasingly understood through a local view, first in organs, then in cells. Ilana Löwy has recounted how cancers were conceptualised as uncontrolled proliferation of cells with local beginning (Löwy, 2013 ). This was, however, not the only way that cancer was characterised at the time. As Löwy writes, Rudolph Virchow and his circle in the mid-19th century agreed on several aspects of how to understand cancer: Cancer derives from normal cells, it relies on blood supply from the host, and it spreads to distant sites of the body. Löwy notes that this conception of the disease suggested a number of different research paths. It could lead to research that investigated vascularisation (as Good’s classification of cancer in a group of morbid blood vessels also indicated) or systemic effects on cancer, i.e. “to develop physiology-centred approaches to malignant disease, and to view cancer as a systemic, not localized, pathology.” These alternative approaches however never became more than “marginal” (Löwy, 2013, pp. 462–463). In the conceptualisation of cancer, its local beginnings and localisation in particular tissues became prioritised, leading to a classification according to a patho-anatomical or topographical dimension. At the same time, the patho-physiological dimension of the disease such as blood supply and constitutional aspects were downplayed.

Diabetes meanwhile proved awkward to categorise on the basis of pathology. There was little in the way of visible lesions in the kidneys, and this despite the clinical characteristics of excessive urination and sweet tasting urine pointing to the kidneys. In 1788 Thomas Cawley performed an autopsy on a diabetes patient and found some change in kidneys, pancreas and liver, but did not link the findings to the disease. Investigating the urine and blood, he focused rather on lost nutrition, when he concluded that expanded tubes of the kidneys letting the nutrients out were responsible for the disease. In general, there was much disagreement on the seat of the disease. Some held it was a disease of the kidneys, others of the liver or the blood (Cawley, 1788, Godtfredsen, p. 474), and this struggle to categories diabetes went on for a century. In 1892, the influential physician William Osler put diabetes with gout under the heading of constitutional diseases, while others categorised it as a kidney disease, and a 1901 textbook described it as a disease “which had no local seat, which is certainly not a disease of the kidney. We therefore place it by itself as a non-febrile general disease, with no ascertained pathology or anatomy“ (Fagge and Pye-Smith 1901 quoted in Tattersall, 2010, pp. 31–32). While physiological approaches to cancer began to be marginalised, they remained central to the investigation of diabetes. The French physiologist Claude Bernard, the main figure in the development of experimental physiology, conducted animal experiments provoking glucosuria and ascertaining more sugar in the blood coming from the liver. In Germany, patho-physiologists such as Carl Wunderlich stated in 1868 that pathology *is* “the physiology of the diseased man” and distanced it from a patho-anatomical focus on localization of lesions in favour of a focus on physiology, biochemistry, and the whole constitution (Faber, 1922, pp. 19–20). According to the pathologist Bernhard Naunyn (1839–1925), the attention to physiology in conjunction with clinical observation, bore fruit “above all and most brilliantly in the pathology of metabolism”, and he complimented German clinical medicine on its work on fevers and diabetes. Incidentally, Naunyn himself was a diabetes researcher pioneering the term acidosis (Faber, 1922, pp. 25–26). Despite the focus on the physiology of the disease, attempts to find the seat or patho-anatomical characterisation of diabetes continued. In 1889 physiological experiments by physician Joseph von Mering and physiologist Oskar Minkowski famously induced diabetes in a dog by removing the pancreas thus indicating that blood sugar is regulated by the pancreas, and at the turn of the century the pathologists Gustave Edouard Laguesse and Leonid Sobolev pinpointed the seat of diabetes to the islets of Langerhans. This patho-anatomical dimension to diabetes, however, did not affect the classification of the disease, which remained physiological.

(c) 20th century

In the first half of the 20th century further investigation, sub-classification and available treatments fixed diabetes and cancer into what we have denoted a patho-physiological track and a patho-anatomical track respectively. The available treatments for cancer – surgery and radiation – were also both localised, and their availability meant that doctors became focused on catching the disease in an early localised state. Subdivision of cancer classifications developed in the 1930’s therefore took the organ of origin as its starting point. This was the case both for clinical classifications which used the anatomical system TNM: Tumour, Nodes involved, and Metastases; and also for the laboratory-based sub-divisions which used microscopic pathology to characterise the tissue and determine the anatomical origin of the cancer (Löwy, 2010, ch. 2). Löwy emphasised that both these systems conceptualised cancer as developing from local to general, and thus placed the onus on discovering the local disease to allow treatment. Chemotherapy which in contrast is a systemic treatment was discovered and developed in the second half of the 20th century, but cancer was by then already stably classified in a patho-anatomical framework according to the position of the primary tumour.

For diabetes, the pancreas was discovered to have a central role, but the focus remained on using pancreatic material to regulate the disease rather than on defining diabetes as a disease of the pancreas.^4^ Pathology did not play a major role for its investigation and classification—physiological experiments did. Likewise while the central treatment of the 20th century is insulin, this treatment regime did not remove focus from maintaining a physiological and biochemical balance. In fact, exercise and diets played central roles alongside the administration of the right doses of insulin. As research has shown, “balance” and regulation remained key concepts and the treatments acted in conjunction with life-style and diet with the aim of keeping the physiology of the patient in balance (Feudtner, 2003; Moore, 2018, 2020; Haalboom, 2024). The treatment options thus co-constituted the disease as physiological, biochemical and dependent on a regulation the whole of the constitution.

Moving back to classifications, the international classification was regularly edited, and in 1949 (ICD-6), the focus of the international classifications underwent a major change from classification of causes of death to also include classification of diseases. Here, Bertillon’s large class of constitutional disease was divided into several classes. Cancers now had their own class, Neoplasms, while diabetes was classed under Allergic, Endocrine System, Metabolic and Nutritional Diseases (World Health Organization, 1949). This class contained diseases that are less easily linked to anatomical sites although some of them are listed under particular glands (thyroid, pituitary etc.). This classification is also conserved in the most recent edition, ICD-11, which we will end the section by discussing.

In the ICD-11, the main structure of the classification is built around different body systems each covered by a chapter e.g.: nervous system, respiratory system, digestive system, etc. These chapters are then subdivided according to different organs-systems. For instance, disorders of the digestive system are classified into diseases of the oesophagus, diseases of appendix, diseases of liver, etc. The class of cancers, Neoplasms, is also subdivided according to anatomic regions: neoplasm of the breast, neoplasm of the urinary tract, neoplasm of digestive tract (oesophagus, stomach, colon, etc.). Cancer thus fits very comfortably into this structure that forefront the patho-anatomical dimension. Meanwhile this central organising principle left out diseases not confined to a system or an organ, such as diabetes. Many of these diseases are gathered under Endocrine, Nutritional, and Metabolic diseases. The common aspect of the diseases under “disorders of metabolite absorption or transport”, “overweight, obesity or specific nutrient excesses”, or “diabetes mellitus” is that they present a regulatory aspect due to the dysfunction of a physiological parameter. For instance, in the case of type 2 diabetes (which was added in ICD-9 from 1979 and is a larger group than type 1 diabetes), the inability for the body to produce or to utilise insulin affects several body parts across the organism: muscles, liver, adipose tissues, brain, etc. As such, diabetes affects a parameter or a function (glucose regulation) that maintains the whole organism, and it is only categorised as organ specific when it comes to the complications.

To conclude, we saw in this section that while diabetes was well-defined in the classificatory systems of the 18th and early 19th century that were based on clinical symptoms and physiological imbalances, it fitted less comfortably into the main structure of classification in the late 19th and 20th century systems where divisions were primarily based on organ systems. Cancer on the other hand was not a unified disease class in the early classifications, but fitted in a more coherent way—as seen from our current perspective – into the 20th century classifications which foregrounded the patho-anatomical dimension. We thus show that different dimensions of the two diseases respectively became prioritised in their classifications: The patho-physiological dimension for diabetes, and the patho-anatomical dimension in the case of cancer. These dimensions are still dominant especially when it comes to diagnosis and standard treatment. Meanwhile other tracks of investigating the diseases were de-emphasized such as the tissue specific origin of diabetes or the vascular processes of cancer.

Adding a historiographical note, the shifts described in the classificatory systems do not follow the traditional medical histories, which for instance place the rise of the patho-anatomical perspective at the Paris hospital in the early 19th century (Ackerknecht, 1967; Jewson, 2005; La Berge & Hannaway, 1998) rather than in the late 19th century. This is because classificatory practices draw on received knowledge and also, as stated earlier, are conservative, while medical history has been interested in discoveries and new practices. A similar delay is found in collection practices (Tybjerg, 2022). The slower and more continuous shifts elucidates better how e.g. cancer was described both according to changes in cells and disturbed flow in the late 19th century (Timmermann 2014, p. 24).

III. Moving across patho-physiological and patho-anatomical classifications.

The development of the classifications of cancer and diabetes is by no means over, and in 2019, the World Health Organization published an overview of the classification of diabetes mellitus. In this publication, the organisation acknowledged the lack of identified characteristics that would help identifying different groups of patients and underlined the need for a refined classificatory system: “None of these characteristics unequivocally distinguishes one type of diabetes from the other, nor accounts for the entire spectrum, of diabetes phenotypes (…) There are several reasons for revisiting the diabetes classification.” (World Health Organization, 2019) This statement is also supported by current researchers working in the field of type 2 diabetes: “Type 2 diabetes is heterogeneous in terms of patient’s clinical presentation, disease course, and response to treatment.” (Udler, 2019) In other words, the level of blood glucose (which is the main way to clinically assess type 2 diabetes) coupled with the absence of auto-antibodies is too broad to account for the heterogeneity of type 2 diabetes cases, underlining the need to identify new criteria (Gale, 2013; Philipson, 2020).

In the field of cancer, similar challenges have been identified. As mentioned in the previous section, the ICD-11 subdivides cancers according to the organ of tumour origin. However, two tumours originating from the same tissue can be extremely different. One common example that has been extensively described in the literature and we will use for the rest of our argument is the case of breast cancer (Redig & McAllister, 2013; Turashvili & Brogi, 2017).

Facing similar difficulties, current research in the two fields have made the same assumption: Established disease classes can be subdivided into sub-types by using molecular markers. Molecular markers are “biological molecules that can be measurable indicators found in the blood, and other body fluids, or tissues by which a particular pathological or physiological process of a condition or disease can be identified” (Das et al., 2017). They can be used for treatment, prognosis, diagnosis but also classificatory purposes (Esteva & Hortobagyi, 2004). In other words, sub-types can be identified within the already existing taxonomic classes (such as breast cancer and type 2 diabetes) according to different molecular markers (Green et al., 2022). This common assumption is explicitly present in the literature in both fields (Dai et al., 2016; Laakso & Fernandes Silva, 2022), and in the rest of this section, we will analyse some of the results obtained using molecular markers to sub-type breast cancer and type 2 diabetes.

- Subtyping breast cancer

As shown in the first section, cancer became a paradigmatic example of a disease classified according to a patho-anatomical dimension: Cancers have been classified based on histopathological analysis according to tumour origin. This dimension is still prioritised in the current classification of neoplasms in the ICD-11. However, since the 1970s, the development of molecular biology and of new technologies allowed the identification of new elements as potentially relevant to refine cancer classes (Weinberg, 2014). More precisely, new molecular markers (such as genetic variants or proteins) have been used to establish molecular profiles of the tumours with the hope to identify new cancer subtypes.

This approach appeared to be relatively successful as detailed molecular profiles (based on different types of molecular markers) have been increasingly used in cancer classification, providing powerful tools for subtyping cancer and selecting treatments: “New technologies are now transforming the field of pathology more rapidly than at any other time during the past 30 years, and it has become increasingly clear that the traditional approach to cancer classification is insufficient.” (World Health Organization, 2023).

In the case of breast cancer, four main subtypes have been identified depending on the status of hormonal and growth factor receptors, including the expression of oestrogen and progesterone receptors and the amplification of the growth factor HER2/neu oncogene product (Reddy et al., 2017; Redig & McAllister, 2013). Based on these criteria, four main sub-types of breast cancer have been identified (triple negative carcinomas, Luminal A carcinomas, Luminal B carcinomas and finally Her-2 positive carcinomas), which have been then associated with different therapeutic recommendations and prognosis (Orrantia-Borunda et al., 2022; Reddy et al., 2017).

Subdivisions however also come with challenges and more recent studies have shown strong overlaps in current cancer classification according to their molecular profiles. Indeed, *The Cancer Genome Atlas Program* (a landmark cancer genomic program that aims at molecularly characterising over 20,000 primary cancers) provided some intriguing results that strongly impacted our understanding of cancer classes and subtypes. First, from a genetic perspective, it has been established that two types of breast cancer (triple negative basal breast carcinomas and luminal breast carcinomas) are as dissimilar from each other as they are from lung cancer (Hoadley et al., 2018). In other words, some triple negative breast cancers are as genetically dissimilar from other subtypes of breast cancers as they are from tumours originating in another organ. These results seem conflicting with the idea that arise from an anatomo-pathological perspective, according to which two subtypes of breast cancers are more similar than tumours originating in different organs. Second, the Cancer Genome Atlas also revealed that tumours from two different organs can share more molecular similarities than tumours originating from the same organ. For instance, some triple negative breast cancers seem to be more similar to some subtypes of lung cancer or serious ovarian cancers (Hoadley et al., 2018). As a consequence, these results do not only suggest the need for stratifying the category of breast cancer as described earlier, but also require a reclassification of cancer, where different cancer categories (for instance some breast cancers and some colon cancers) could be grouped together based on similar patterns, thus challenging the prioritisation of the patho-anatomical dimension (Tomczak et al., 2015; Green et al., 2022).

More generally, these findings allow the identification of “pan-cancer clusters” and underline the tensions between on one hand a classification based on a patho-anatomical dimension, and on the other hand, a molecular profile-based classification (Plutynski, 2019). As a result, Hoadley and colleagues claimed that: “Anatomic classification should be supplemented by a classification system based on molecular alterations, shared by tumours across different tissues” (Hoadley et al., 2018). According to the authors, approximately 10% of cases would be reclassified according to this molecular classification relative to the current patho-anatomical classification. This reclassification could significantly increase the predictive clinical power regarding therapeutic outcomes (Hoadley et al., 2014, 2018).

The use of molecular profiles to refine cancer classes thus challenges the patho-anatomical classification. The process of sub-typing, strongly oriented by molecular profiling of the tumour, reveals cross-cutting subtypes regardless of the patho-anatomical dimension of the tumour. Those cross-cutting subtypes suggest molecular variants that play a key role in cancers across multiple organs, and therefore cause problems both for the patho-anatomically confined understanding of cancer and its classification.

(b) Subtyping type 2 diabetes

In the historical section, we have shown that compared to cancer, diabetes was primarily classified according to a patho-physiological dimension and this is true of type 2 diabetes to an even larger extent. In the ICD-11, type 2 diabetes (like the other types of *diabetes mellitus*) falls under the endocrine disease category and is defined as: “Diabetes mellitus type 2 (formerly non-insulin-dependent diabetes mellitus (NIDDM) or adult-onset diabetes) is a metabolic disorder that is characterised by high blood glucose in the context of insulin resistance and relative insulin-deficiency.” (ICD-11 for Mortality and Morbidity Statistics, Version: 01/2023) Interestingly, in clinical practice, type 2 diabetes diagnosis is mainly an exclusion diagnosis: a case of diabetes that is not diabetes mellitus in pregnancy, nor other specified type of diabetes mellitus (such as the Maturity Onset Diabetes of the Young MODY), nor a case of idiopathic type 1 diabetes mellitus. Just like in the field of cancer, the development of new technologies and the possibility to identify molecular markers is regarded as a promising path to identifying sub-types of type 2 diabetes.

As mentioned previously, one main difference between type 2 diabetes and cancer is that type 2 diabetes is characterised by a dysfunction and not by the presence of lesions in a specific organ. However, current research in type 2 diabetes now orientates towards a stronger organ anchoring for understanding the disease. Indeed, it suggests that different sub-types of type 2 diabetes could be organ or tissue specific (Miguel-Escalada et al., 2019): Different organs could play different roles in different type 2 diabetes subtypes and explain the clinical heterogeneity of the disease. Indeed, specific organs (liver, muscles, brain, etc.) play different roles in the blood glucose regulation suggesting different aetiologies that could be organ related (Xourafa et al., 2024). Moreover, diabetic patients display an important heterogeneity in the evolution of the disease. Indeed, type 2 diabetes has a progressive evolution that can slowly affect the heart, the liver, the ocular system, or the neurological system. Therefore, there is also a tissue specific risk progression among the patients deemed worth exploring. So while the patho-anatomical division of disease progression is included in the ICD-11, research now moves towards associating it with origins of the disease that are also tissue-specific.

The overall aim of this new line of research is to produce a map of key diabetes-relevant tissues and study tissue specific genetic variations in order to better understand not only their impact on whole-body physiology, but also their local impact on organ-specific genetic regulation and transcription (McCarthy, 2017). For instance, some genetic variants having an impact in type 2 diabetes have been identified as exerting their effects exclusively in the adipose tissue (McCarthy, 2017). The aim of current research is thus partly to move away from a characterization of the disease that is at the whole organism level, and to be able to identify more precisely *“where”* type 2 diabetes is anchored in terms of specific tissues or cell types dysfunctions.

To sum up, diabetes has historically been understood most readily as a patho-physiological disease (based on its functional dimension), but current efforts to subtype type 2 diabetes try to anchor the disease into specific organs to discriminate between different groups of patients based on disease development and evolution.

By comparing the two fields, it appears that results from current research suggest that focusing on either the patho-anatomical or the patho-physiological dimensions of a disease to establish classification falls short. The use of molecular markers however reopens dimensions for characterising cancer and type 2 diabetes that have been downplayed in the course of their classificatory history. We thus show how new approaches such as molecular and genetic profiling move across the major disease groups of type 2 diabetes and cancer. On one hand, cancer was streamlined and gathered in one class prioritising the patho-anatomical dimension, but current research stresses the importance of cross-organ similarities and intra-organ dissimilarities, weakening the prioritisation of anatomic site to classify cancer. On the other hand, diabetes classification has prioritised patho-physiological dimension, but current research tries to map the different subtypes of type 2 diabetes onto different tissues. Molecular markers represent a new departure for cancer and type 2 diabetes classification acting as a crossing point into dimensions of the diseases that have been downplayed in the historical classifications.

IV. Opening dimensions and new challenges for modern classifications.

The previous sections showed that in the course of the history of classification some dimensions have been emphasized while others have been downplayed. As mentioned before, this priorisation strongly frames and defines what will be under investigation and how, leading to some form of conceptual inertia. However, the recent development and use of molecular markers reintroduces aspects that have been downplayed in the course of the history of classification: Cancer is increasingly studied and classified through the unravelling of processes affecting several organs and challenging the prevalence of the patho-anatomical dimension for classificatory purposes; and conversely, type 2 diabetes is thought to encompass different tissue specific mechanisms differentiating patients, and thus underlining the importance of exploring the patho-anatomical dimension of this patho-physiological disease^5^. In the last section, we would like to draw some conclusions from this comparison: first, we want to emphasise why looking back matters to understand the current evolution of disease classification and second, we want to present some of the challenges that arise; or re-emerge when more dimensions are brought into play.

The history of classification showed that cancer and diabetes were harder to classify according to some dimensions than others, but the dimensions of the diseases not highlighted do not completely disappear. In the field of cancer, the patho-anatomical dimension was the dominant one. This perspective had a strong influence and practical consequences in the field of oncology. For instance, therapeutic strategy and disease management are still strongly defined by and designed according to the tissue of origin of the primary tumor, making cases of cancers of unknown primary sites particularly difficult to manage (Olivier et al., 2021). Another consequence of this dimension being dominant is the way clinical oncologists become specialized in specific tumor types (Chen et al., 2021). However, the use of biomarkers challenges this traditional organization of oncology around a tissue-based understanding of the disease. As some authors underline, the emergence of a “tissue agnostic” approach in oncology represents an important shift (Carbone, 2020; Mansinho et al., 2023).

Indeed, this theoretical shift has been experimentally explored and appears (at least partly) to be supported by the use of biomarkers to point back to dimensions that were explored e.g. in the 19th century, but which were marginalised as the diseases were later more exclusively characterised by the patho-anatomical dimension (Löwy, 2013). Indeed, this is partly due to the identification of molecular markers and functional pathways that cross over the organ classification (as described in the previous section). This, in turn, has important consequences for cancer classification and management. Indeed, this molecular re-classification can be used to determine treatments that target molecular alterations and driving pathways that are common across different subtypes of cancer (lung, breast, gastrointestinal, skin and bone marrow malignancies) (Hoadley et al., 2018). One example is the targeting of the development of new blood vessels in tumours (a feature of cancer noted both in Cullen’s classification of morbid blood vessels and 19th research). This is achieved with the anti-angiogenic treatment, Bevacizumab. As the development of tumour-vasculature has become an important - functional - feature in many different cancer types, regardless of their patho-anatomical characterisation, bevacizumab indication has been extended to metastatic breast cancer, non-small cell lung cancer, glioblastoma, renal cell carcinoma, ovarian cancer and cervical cancers (Garcia et al., 2020). This example shows an interest in focusing on the patho-physiological dimension of tumours. Indeed, tumours originally classified into different groups according to their patho-anatomical dimension can be gathered together under a common therapeutic strategy that targets a common functional pathway (for instance vascularisation). This reasoning has now been extended through the concept of repurposing drugs. Repurposing drugs are drugs that have been approved for a specific condition and are used for other diseases: “If two diseases where same pathways or signalling is affected, the drugs that target the specific pathway can be used in both of diseases irrespective of their structural dissimilarity.” (Aggarwal et al., 2021). This tends to become more and more common, especially with the development of “basket trials”, which test one treatment against different tumours that share a particular molecular defect, regardless of the tissue of origin (Carbone, 2020).

This new approach opens exciting avenues, especially for cancers that are unresponsive or resistant to treatments (Qin et al., 2019). Indeed, even if a classification according to patho-physiological dimensions that can be targeted by particular therapies is not yet in place, it is definitely one of the expectations that come with the development of molecular biology: “Molecular–genetic profiling can be used to refine existing classifications of tumours and, for a small but increasing number of cancers, even determine the treatment irrespective of histotype.” (Carbone, 2020) In this sense, cancer classification might be improved by taking into account common functional pathways that can be targeted by common treatment, and thus opening up again to the patho-physiological dimension to complement the patho-anatomical classification.

Similarly in diabetes, the historical development led to the establishment of the current dominant view of the disease as a general imbalance of the body instead of a disease associated with a specific organ. This dominant perspective is actually endorsed by the main scientific and medical institutions, despite acknowledged limitations: “For type 2 diabetes, the definition and classification of the disease remains rudimentary. The disease is currently defined by the concentration of blood glucose.” (Narayan et al., 2023) In this context, the development of new technologies (such as molecular omics measurements) are perceived as an opportunity to: “use Thomas Kuhn’s terminology, a paradigm shift away from normal science, by redefining how we investigate the fundamental biology of the disease.”^6^ (Narayan et al., 2023)

This shift echoes what we described in the historical section II, when diabetes in the 19th century was characterised variously as a disease of the liver, of the kidney or of the pancreas despite the lack of clear lesions. Now, multi-omics studies have refined our understanding of type 2 diabetes and drawn regulatory maps for key diabetes-relevant tissues to identify the effects of genetic variants on component pathways in a tissue-specific manner (McCarthy, 2017; Miguel-Escalada et al., 2019; Pearson, 2019). Following the assumption that type 2 diabetes pathogenesis results from inter-organ interactions, attempts were made to propose to use markers to understand the progression and risk complications associated with different type 2 diabetes subtypes (Xourafa et al., 2024). Although the use of genetic and molecular information is less advanced in diabetes than in cancer, some interesting new perspectives open up with investigating the patho-anatomical dimension. For instance, the project AMPT2DK offers a collaborative platform to combine genetic data with “with information on gene expression and epigenomics in relevant tissues, and clinical information, (to) provide clues about the effects of genetic changes within an individual’s genome that increase or decrease one’s risk of developing type 2 diabetes and its complications, including heart and kidney disease^7^.”

Indeed, type 2 diabetes is associated with several diseases affecting different organs, such as vascular diseases (atherosclerosis, infarctus, etc.), retinopathia, nephropathia, hepatic disease or even skin diseases. Therefore, molecular and genetic information have been used to stratify patients in order to predict which patients are most at risk of developing a disease affecting one organ rather than the other. For instance, diabetic kidney disease (DKD) remains a significant cause of mortality in patients with diabetes. However, the detection of DKD is mostly based on the level of albumin in the patient biofluid, which is often associated with a late diagnosis and therefore with a worse prognosis. Based on new technologies (such as spatial transcriptomics or single-cell RNA), it has been possible to differentiate between the healthy and disease states of kidney cells (Kondo et al., 2024). These technologies have then allowed researchers to identify cells in different molecular states across tissue samples (Kondo et al., 2024). Preliminary studies have then shown that, using a relatively small set of proteins, it is possible to discriminate among kidney samples from patients with diabetes mellitus, those who do not present kidney disease signs and those with stage III DKD. In other words, it allows, based on a tissue sample analysis, to determine early on the disease trajectory of patients with diabetes mellitus. Such studies reflect the growing interest in using histological molecular markers and cellular composition to stratify diabetes patients based on different associated disease risks (Kondo et al., 2024).

In this sense, as two main dimensions have co-existed in the history of type 2 diabetes research, dissection of pathological mechanisms in specific organs and examination of fluid regulation, it seems that while the latter was established as the dominant characterization for classificatory purpose for a while, part of the scientific community in diabetes is currently advocating in favor of a renewal of interest for the former (Kondo et al., 2024).

Finally, it is worth mentioning the use of molecular markers to bring dimensions of diseases to the fore that have previously been downplayed comes with its own classificatory problems. In the field of cancer, the literature (both in philosophy and biology) established that the increasing use of molecular markers to refine cancer classification is now facing important challenges: “Initially, researchers investigating cancer genomics seemed to think such issues might be resolved by molecular “subtyping”. However, genomic sequencing and its sequelae – transcriptomics, proteomics, epigenomics, etc.—have if anything, complicated cancer classification (…).” (Laplane & Plutynski, 2023). This complexification is due to two main challenges: first, the increasing overwhelming amounts of markers that can be used to subtype complex diseases, and second some of the characteristics of complex diseases such as redundancy, pleiotropy, polygenic causes, and low penetrance make it harder to pinpoint which biomarkers should be prioritised for subtyping (Bertolaso, 2016; Plutynski & Laplane, 2023). Using biomarkers thus presents us with some of the same difficulties of choosing a main dimension that also troubled earlier classification systems. As Farr noted about classifying based on symptoms versus classifying based on localisation of pathological lesions: The same symptom may be associated with several underlying diseases.

Indeed, the biomedical field is now witnessing an increasing amount of literature identifying potential relevant molecular markers. This is currently the case in both, type 2 diabetes, and breast cancer research. In the case of breast cancer, there has been an increasing number of potential subtypes that not only coexist with each other, but also might be contradictory with one another (Plutynski, 2018). If in type 2 diabetes the use of molecular markers to improve disease classification is not as advanced as in cancer, one can already witness a proliferation of different types of molecular markers that could be used to subtype type 2 diabetes leading to a proliferation of potential sub-classifications of type 2 diabetes (Deutsch, 2022). As a result, it becomes difficult in both fields to identify and assess which of the potential biomarkers are to be prioritized for classificatory purposes (Green, 2021; Green et al., 2022; Plutynski & Laplane, 2023).

These challenges have been extensively studied in the field of cancer by both philosophers (Bertolaso, 2016; Yvonnet et al., 2019; Plutynski & Laplane, 2023) and scientists (Weinberg, 2014), and it is likely that these analyses also apply to type 2 diabetes. The identification and justification of which factors should be disregarded or prioritized for classificatory purposes is therefore not solved by biomarkers but rather restated in new ways.

## Conclusion

We set out to explore the possibilities and limitations arising from current sub-classification and re-classification of cancer and diabetes in the light of how these complex diseases have been classified and understood in the past. We argue that because classification requires particular dimensions of diseases to be prioritised—such as the patho-anatomical or patho-physiological dimensions—then characteristics related to other aspects are marginalised.

First, we traced the history of how cancer and diabetes have been classified. We emphasised how different classificatory schemes created tensions for the two diseases. Cancer was spread across local, general, flow related and blood-related classes in early classification systems, while diabetes in the 20th century did not easily lend itself to the patho-anatomical logic of the main classification system, and its complications in different body parts became the main way of subcategorizing it. The diseases became classified according to two different dimensions: the patho-anatomical dimension for cancer (being characterised by the presence of a pathological lesion in the organ or tissue of origin) and the patho-physiological dimension for diabetes (being characterised by a physiological unbalance or dysfunction). This meant that for diabetes its connection to particular organ systems was marginalized and for cancer physiological approaches were downplayed as were similarities between cancers originating in different organs. The conservative nature of classifications means that the historical approaches to diseases remained enfolded in the classifications—stressing only some aspects of diseases while others were downplayed or marginalized. At the same time, these classifications had a strong influence in the scientific and medical organization of oncology and diabetology, creating a form of conceptual inertia in the way both diseases were understood and investigated.

Second, we showed how the patho-anatomical and the patho-physiological characterization of cancer and diabetes became increasingly unsatisfying, and the development and the use of molecular markers for classificatory purposes challenged the way dimensions have been historically prioritized and shaped classifications. Indeed, molecular markers are becoming associated with underlying physiological dysfunction displaying a systemic aspect in cancer, and with organ and tissue specific pathological mechanisms in diabetes. We thus emphasise that molecular biology and genetics allows the crossing of hitherto prioritised dimensions. New potentials for understanding disease have been found by *crossing classificatory paths*: cancer researchers consider regulatory and physiological aspects, and diabetes researchers are becoming interested in organ and tissue specific aspects of diabetes. This constitutes an opening of several dimensions akin to the situation in the 19th century when cancer and diabetes were investigated according to several dimensions, as well as an important shift in the understanding of cancer and diabetes.

So while disease classification systems to many researchers might seem like an activity far downstream from the sophisticated stratifications of research, we argue that classifications and their histories affect research by defining the salient characteristics - or dimensions - of the disease and sidelining others. It also indicates that looking across non-prioritised dimensions may be productive. Like cancer research, diabetes research is now beginning to look beyond the patho-physiological way diabetes was defined and understood, and like cancer it might be sub-categorised by its origins as well as by its complications.

With this work, we methodologically draw together historical and philosophical approaches to understand tensions arising in the classification of complex disease both historically and today. We historicise the philosophical idea of classificatory dimensions, and we conceptualise historical developments in understandings of disease as shifts in the main dimensions of disease classification. We argue that—if combined—philosophy and history can contribute to making explicit what the consequences of classifications always leaving some dimensions out are, and thus cast light on some of the underlying assumptions in today’s research on cancer and diabetes, and on the histories enfolded in our classificatory systems.

## Footnotes

1 Even if it makes good historical sense to view symptoms as a separate dimension, we decided that proposing a new conceptual framework around dimensions would extend beyond the scope of this paper. We therefore used a framework close to Nordenfelt’s, where symptoms can fit under both anatomical (such as lump in the breast) or physiological (such as an excess of urination).

2 Our distinction between patho-physiological and patho-anatomical recalls the famous distinction by Owsei Temkin between ontological and physiological aspects of disease (Temkin, 1977). As Temkin did, we underline that different aspects of the same disease (patho-physiological or patho-anatomical) can be prioritized, but that none of them is enough on their own. We do, however, not follow Temkin’s distinction between different natures of disease as either general entities or individual deviations.

3 In Sauvages’ later nosology *Nosologia methodica* 1768 the similar category of flows that are not blood or is called “lasting flows”.

4 Diabetes was never classified as a disease in the patho-anatomical sub-class of “diseases of the pancreas”, which included for instance inflammation, cysts or fibrosis of the pancreas. In ICD 4 (1929) and ICD 5 (1938) diabetes is under “general diseases”, while diseases of the pancreas are listed under “diseases of the kidneys and anexa” in ICD 4 (1929) and in ICD 5 (1938) under “diseases of the pancreas (other than diabetes)”. The latter acknowledges the link between diabetes and the pancreas, but still does not include it.

5 We do acknowledge that other approaches than the molecular one are employed in both fields in an attempt to reduce clinical heterogeneity (Alhqvist et al., 2019). However, in both fields, the search for molecular markers became one of the most common approaches.

6 We quote the Kuhnian reference and vocabulary used by the authors (Narayan et al., 2023) even if we do not suggest a Kuhnian analysis in this article, but a change of dimensions, which in contrast to paradigms can be compared even if they frame the understanding of disease.

7 From the webpage of the project Type 2 Diabetes Knowledge Portal - *About | Type 2 Diabetes Knowledge Portal*. (s. d.). Last consulted on November 28th November 2025, https://t2d.hugeamp.org/about.html.
